# Aging Impairs Cerebrovascular Reactivity at Preserved Resting Cerebral Arteriolar Tone and Vascular Density in the Laboratory Rat

**DOI:** 10.3389/fnagi.2019.00301

**Published:** 2019-11-08

**Authors:** Armand R. Bálint, Tamás Puskás, Ákos Menyhárt, Gábor Kozák, Imre Szenti, Zoltán Kónya, Tamás Marek, Ferenc Bari, Eszter Farkas

**Affiliations:** ^1^Department of Medical Physics and Informatics, Faculty of Medicine, University of Szeged, Szeged, Hungary; ^2^Department of Physiology, Faculty of Medicine, University of Szeged, Szeged, Hungary; ^3^Department of Applied and Environmental Chemistry, Interdisciplinary Excellence Centre, University of Szeged, Szeged, Hungary; ^4^MTA-SZTE Reaction Kinetics and Surface Chemistry Research Group, University of Szeged, Szeged, Hungary

**Keywords:** aging, cerebral blood flow, cerebral ischemia, neurovascular coupling, arteriolar diameter, reperfusion, spreading depolarization, vascular density

## Abstract

The age-related (mal)adaptive modifications of the cerebral microvascular system have been implicated in cognitive impairment and worse outcomes after ischemic stroke. The magnitude of the hyperemic response to spreading depolarization (SD), a recognized principle of ischemic lesion development has also been found to be reduced by aging. Here, we set out to investigate whether the SD-coupled reactivity of the pial arterioles is subject to aging, and whether concomitant vascular rarefaction may contribute to the age-related insufficiency of the cerebral blood flow (CBF) response. CBF was assessed with laser-speckle contrast analysis (LASCA), and the tone adjustment of pial arterioles was followed with intrinsic optical signal (IOS) imaging at green light illumination through a closed cranial window created over the parietal cortex of isoflurane-anesthetized young (2 months old) and old (18 months old) male Sprague–Dawley rats. Global forebrain ischemia and later reperfusion were induced by the bilateral occlusion and later release of both common carotid arteries. SDs were elicited repeatedly with topical 1M KCl. Pial vascular density was measured in green IOS images of the brain surface, while the density and resting diameter of the cortical penetrating vasculature was estimated with micro-computed tomography of paraformaldehyde-fixed cortical samples. Whilst pial arteriolar dilation in response to SD or ischemia induction were found reduced in the old rat brain, the density and resting diameter of pial cortical vessels, and the degree of SD-related oligemia emerged as variables unaffected by age in our experiments. Spatial flow distribution analysis identified an age-related shift to a greater representation of higher flow ranges in the reperfused cortex. According to our data, impairment of functional arteriolar dilation, at preserved vascular density and resting vascular tone, may be implicated in the age-related deficit of the CBF response to SD, and possibly in the reduced efficacy of neurovascular coupling in the aging brain. SD has been recognized as a potent pathophysiological contributor to ischemic lesion expansion, in part because of the insufficiency of the associated CBF response. Therefore, the age-related impairment of cerebral vasoreactivity as shown here is suggested to contribute to the age-related acceleration of ischemic lesion development.

## Introduction

The structural and functional integrity of the cerebral microvascular system is essential for optimal neural function. It has been long documented that the cerebral arteriolar tree, capillary bed, and venous circulation may undergo (mal)adaptational structural rearrangement with aging (Farkas and Luiten, 2001; Riddle et al., 2003; Bogorad et al., 2019; Fulop et al., 2019; Kalaria and Hase, 2019). Cerebral blood flow (CBF) at rest appears to be lower in the aging brain (Farkas and Luiten, 2001; Riddle et al., 2003; Kalaria and Hase, 2019), and the reactivity of the cerebral microvasculature to neuronal activation or hypercapnic challenge becomes less efficient, partly because of an increased burden of oxidative stress (Riecker et al., 2003; Park et al., 2007; Mayhan et al., 2008; Toth et al., 2014; Balbi et al., 2015). Weakening neural control, particularly the loss of cholinergic innervation of cortical microvessels from the basal forebrain, or inadequate noradrenergic signaling from the locus coeruleus may also account for failing vasoregulation at old age (Farkas and Luiten, 2001; Lecrux and Hamel, 2016; Lecrux et al., 2017; Nizari et al., 2019). Further, the neurovascular unit and blood-brain barrier function has been found to be subject to molecular and biochemical alterations with aging (Cai et al., 2017; Erdő et al., 2017; Osipova et al., 2018). Collectively, these age-related modifications of the cerebral microvascular system have been implicated in cognitive impairment (Farkas and Luiten, 2001; Di Marco et al., 2015; Toth et al., 2017) and worse outcomes after ischemic stroke with increasing age (Ay et al., 2005; Popa-Wagner et al., 2007; Faber et al., 2011; Ma et al., 2019; Zhang et al., 2019).

Spreading depolarization (SD) poses a cerebrovascular challenge, and is caused by a local supply-demand mismatch in the cerebral gray matter (Ayata and Lauritzen, 2015; von Bornstädt et al., 2015). Acute cerebral ischemia—focal or global—readily triggers SD, and persisting ischemic conditions favor the recurrence of SD events (Hartings et al., 2003, 2017). SD represents a near-complete breakdown of the resting membrane potential of a critical mass of neurons, which propagates over the injured cerebral cortex at a slow rate (2.8 mm/min; Mayhan et al., 2008; Dreier and Reiffurth, 2015). Ideally, SD is coupled with a prominent CBF response including a substantial hyperemic element to support the restoration of resting membrane potential. Sufficient nutrient supply afforded by hyperemia is converted to ATP to be used by energy-dependent ion pumps (Ayata and Lauritzen, 2015). However, the hyperemic component of the CBF response to SD becomes insufficient in brain tissue being challenged by ongoing ischemia (Dreier, 2011; Hoffmann and Ayata, 2013; Hartings et al., 2017), and may be compromised further by aging (Farkas and Bari, 2014; Menyhárt et al., 2015; Hertelendy et al., 2019). The delayed or obstructed delivery of nutrients postpones or altogether aborts repolarization after SD (Dreier, 2011). Importantly, the increasing cumulative duration of recurrent SD events or irreversible depolarization from a single event may prove lethal to the tissue (Dreier et al., 2006; Dreier, 2011), because high intracellular Ca^2+^ content sustained with SD, accumulating partly through NMDA receptors and voltage-gated Ca^2+^ channels, leads to excitotoxic damage (Hartings et al., 2017; Reinhart and Shuttleworth, 2018; Szabó et al., 2019). Experimental and clinical evidence taken together have recently led to the formulation of the opinion that SD represents a universal principle of cortical lesion development (Hartings et al., 2017).

We have repeatedly demonstrated in anesthetized, middle-aged and old rodents, that the hyperemic response to SD is blunted with aging, while an early vasoconstrictive component in the CBF response becomes more apparent in the old compared to the young brain (Farkas et al., 2011; Clark et al., 2014; Menyhárt et al., 2015, 2017b). On the basis of our experimental data, we have postulated that ischemic lesion progression may be accelerated by aging, because the metabolic need caused by SDs that occur and propagate over the ischemic penumbra is met by an inefficient flow response. This, in turn, is expected to cause a persistent supply-demand mismatch that delays repolarization from SD, and ultimately leads to neurodegeneration (Farkas and Bari, 2014; Menyhárt et al., 2015).

In the service of our research efforts to tackle the coupling between SD and the associated CBF response, we have developed an experimental, multi-modal wide-field optical imaging system that offers an appropriate spatio-temporal resolution for studying SD (Farkas et al., 2010). The combination of two of the synchronous modalities, laser-speckle contrast analysis (LASCA) and intrinsic optical signal (IOS) imaging at green light illumination offers a unique opportunity to examine CBF changes synchronous with variations of pial arteriolar caliber, and pial arteriolar architecture (Farkas et al., 2010; Bere et al., 2014). Indeed, in addition to the SD-related CBF variation, the hemodynamic response propagating with SD is obvious at the level of the pial and penetrating arterioles (Leão, 1944; Brennan et al., 2007; Ayata and Lauritzen, 2015; Unekawa et al., 2017; Menyhárt et al., 2018).

Here, we further explored our working hypothesis that age weakens the hemodynamic response to SD—a contributing factor to accelerated lesion progression after ischemic stroke. We set out to evaluate whether the dilation of pial arterioles in response to SD is impaired by aging. We conducted our investigation in the intact cerebral cortex, under ischemia, and during reperfusion. In order to evaluate whether a potential age-related insufficiency of the hemodynamic response has any quantifiable vascular anatomical correlate, we also analyzed the density and resting diameter of the pial vascular network, and the density and vascular caliber of the cortical penetrating vessels in perfusion-fixed brain tissue. Finally, the CBF maps generated in our experiments were evaluated to understand whether the spatial pattern of flow distribution in the cerebral cortex was subject to age.

## Materials and Methods

The experimental procedures were approved by the National Food Chain Safety and Animal Health Directorate of Csongrád County, Hungary. The procedures were performed according to the guidelines of the Scientific Committee of Animal Experimentation of the Hungarian Academy of Sciences [updated Law and Regulations on Animal Protection: 40/2013. (II. 14.) Gov. of Hungary], following the EU Directive 2010/ 63/EU on the protection of animals used for scientific purposes and reported in compliance with the ARRIVE guidelines.

### Experimental Procedures—*in vivo* Functional Imaging

Experimental procedures and animals were identical to those reported previously (Menyhárt et al., 2017b). Briefly, a closed cranial window (4.5 × 4.5 mm) incorporating an intracortical local field potential (LFP) glass capillary electrode at its lateral edge was created over the right parietal cortex of isoflurane-anesthetized young adult (2 month-old, *n* = 11) and old (18–20 month-old, *n* = 11) male Sprague–Dawley rats. The cranial window was continuously transfused with artificial cerebrospinal fluid (aCSF). IOS at an illumination centered on the isosbestic point of hemoglobin (light-emitting diode of 530 nm peak wavelength; SLS-0304-A, Mightex Systems, Pleasanton, CA, USA; with a bandpass filter 3RD 540–570 nm, Omega Optical Inc., Brattleboro, VT, USA) was acquired in combination with the simultaneous monitoring of CBF by LASCA (laser diode HL6545MG, Thorlabs Inc., Newton, NJ, USA; 120 mW; 660 nm emission wavelength) at a resolution of 1,024 × 1,024 pixels (pixel size: 3.71 μm). Green IOS and LASCA images were captured alternately, each at 1 Hz frequency. The green IOS image sequences were used off-line to estimate the vascular density of the pial surface in the field of view, and to measure diameter changes of pial arterioles over the experimental protocol. LFP was acquired and filtered in DC mode to confirm SD occurrence, exactly as detailed previously (Menyhárt et al., 2017b).

The experimental protocol consisted of three subsequent phases: baseline (i.e., intact cerebral perfusion), ischemia, and reperfusion, each lasting approximately an hour. Incomplete, global forebrain ischemia and later reperfusion were induced by the bilateral occlusion and later release of both common carotid arteries. Experiments were terminated by the overdose of isoflurane. During each phase of the experiments (i.e., baseline, ischemia and reperfusion), 3 SDs were elicited with 1–3 μl 1 M KCl, injected to the brain surface at the medio-caudal corner of the cranial window, at an inter-SD interval of 15 min.

### Experimental Procedures—*ex vivo* Structural Imaging

Micro-computed tomography (micro-CT) scanning was implemented to generate 3D images of the penetrating vascular architecture and characterize its density in tissue samples of the parietal cerebral cortex. Additional young (*n* = 7) and old (*n* = 7) male Sprague–Dawley rats were transcardially perfused with 4% paraformaldehyde in deep isoflurane anesthesia, and the brains were removed, post-fixed and stored in the same solution at 4°C until further processing. Iodine was selected as a contrasting agent to enhance the contrast between solution-filled vascular lumina and the surrounding tissue since their discrimination in raw CT images would be problematic due to the similarity of their attenuation coefficients. Small cortical tissue blocks from the frontoparietal cortex (primary motor/somatosensory area) were dissected and transferred to Lugol’s iodine solution (KI + I_2_; 2 g KI and 1 g I_2_ in distilled water of a final volume of 100 ml) to be incubated for 48 h at room temperature. The samples were rinsed in distilled water for 10 min, and embedded in 1% agarose gel in custom-tailored 1 ml plastic pipette tips, which were then sealed with Parafilm. This procedure results in iodine enrichment of the tissue, in which lumina of blood vessels corresponded to spaces devoid of the contrasting agent. Note that the procedure does not discriminate between arterioles and venules.

CT scanning was conducted with a Bruker Skyscan 2211 micro-CT scanner (Bruker, Brussels, Belgium). Raw scans were acquired at a voxel size of 2.5 μm, with a mean acquisition time of 4 h. The exposure time was set at 525 ms, at a voltage of 100 kV and a current of 700 μA, with a rotation step of 0.1°. The projection data were corrected for distortion and reconstructed by correcting the ring artifacts. Reconstructed isotropic voxel size was 15.625 μm^3^. Images were reconstructed using the scanner software (NRecon 1.6.6.0, Skyscan, Brucker micro-CT, Belgium) and converted to BMP images for further processing and analysis in MatLab (Mathworks, Natwick, MA, USA; Figure 2A).

**Figure 1:**
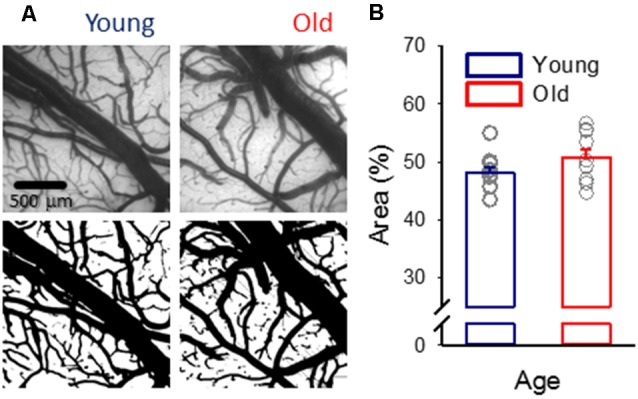
Pial vascular density. **(A)** Representative images (top) of the pial surface of a young and an old preparation at green light illumination (orientation: medial to the top, rostral to the right). The relative area covered by vessels was calculated in computed images (bottom). **(B)** Vascular density was expressed as the area covered by the pial vascular network relative to the full image size. Individual values are gray symbols; bars show mean ± SEM (*n* = 11/10, young/old). An independent samples *T*-test was used to statistically evaluate age-related differences (*p* < 0.112).

**Figure 2:**
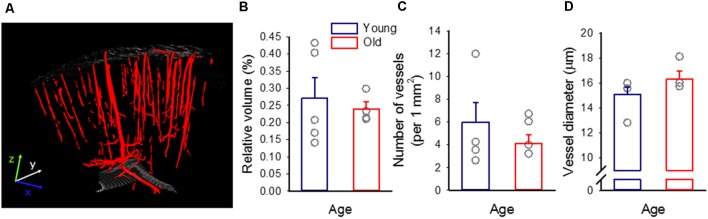
The density of the cerebrocortical penetrating vasculature. **(A)** A representative 3D micro-CT reconstruction of the penetrating vascular architecture of the frontoparietal cortex of a young preparation. **(B)** Tissue volume filled by blood vessels, relative to the size of the sample. **(C)** The mean number of vascular profiles in the sample. **(D)** The diameter of vascular profiles encountered. Individual values are gray symbols; bars show mean ± SEM (*n* = 5/4, young/old). A one-way analysis of variance (ANOVA) was used to statistically evaluate age-related differences (*p < 0.05). No statistically significant difference has been found among any of the variables investigated.

### Signal Processing and Data Analysis—*in vivo* Functional Imaging

Green IOS images provide a sharp contrast between pial vessels (high green light absorption by hemoglobin) and the cortical parenchyma (low green light absorption by the nervous tissue), which enables reliable automated edge detection to estimate pial vascular density and changes in pial arteriolar diameter (Farkas et al., 2010).

The density of the pial vasculature in the field of view was analyzed in MatLab and was expressed as the relative area covered by vessels with respect to the entire cortical surface under analysis (Figure 1). The first green IOS frame was taken in each experiment. After smoothing (15 × 15 pixel square box filter), the image was rotated by 0, 11.25, …, 90 degrees, and edges were detected line by line along the horizontal and vertical axes. Vessels were considered if local minima and local maxima of the first spatial derivative corresponding to the putative edges were detected within 100 pixels. Vessels, which were found in all rotated images were included. In a second approach, the smoothed images were segmented into vessel—no vessel areas by simple thresholding. Threshold intensity was manually determined for each animal. For a third approach, vessel contours were detected by applying a Canny edge detection on raw images. Finally, the outcome of these three approaches were merged and manually adjusted, if necessary. Vascular density was estimated in an 1,800 × 1,800 μm area centered on the field of view.

**Figure 3:**
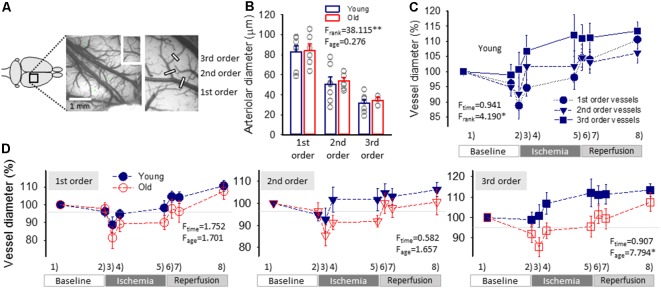
Pial arteriolar diameter between subsequent events of the experimental protocol. **(A)** A green intrinsic optical signal (IOS) image of a representative young preparation demonstrates the ranking of pial arterioles. **(B)** The Resting diameter of 1st, 2nd and 3rd order pial arterioles in the two age groups. Individual values are gray symbols; bars show mean ± SEM (*n* = 7/6, 7/7 and 7/3, young/old, 1st, 2nd and 3rd order). A multivariate ANOVA paradigm was used for statistical analysis, considering vessel rank or age as factors (**p < 0.01). **(C)** Changes of the pial arteriolar diameter of 1st, 2nd and 3rd order arterioles relative to baseline (100%) over the experimental protocol. Sampling times are: (1) baseline—prior to the elicitation of the first spreading depolarization (SD); (2) baseline—prior to ischemia induction; (3) ischemia—minimum vascular diameter after ischemia onset; (4) ischemia—prior to the elicitation of the first SD under ischemia; (5) ischemia—prior to the initiation of reperfusion; (6) reperfusion—maximum vascular diameter after reperfusion initiation; (7) reperfusion—prior to the elicitation of the first SD under reperfusion; and (8) reperfusion—prior to the termination of the experiment. **(D)** The impact of age on the relative changes of pial arteriolar diameter of 1st, 2nd and 3rd order arterioles. Data are given as mean ± SEM (*n* = 8/5, young/old). In panels **(C,D)**, a repeated measures paradigm was used for statistical analysis, considering vessel rank or age as factors (*p < 0.05 and **p < 0.01).

In order to measure changes in arteriolar diameter, we refined a method previously validated in our lab (Farkas et al., 2010). Three arterioles in their order of branching were selected in the field of view for the analysis. A rectangular region of interest (ROI) was manually positioned on the selected arteriolar segments. A segment of the largest caliber arteriole entering the field of view from the frontal direction was taken before its first branching (first-order vessel). Next, its first branch arteriole (second-order vessel), and a lower order branch (third-order vessel) were analyzed (Figure 3A). The analysis script written in a MatLab environment first detected and corrected tissue swelling-related movement artifacts by a 2D cross-correlation based algorithm, and then calculated vessel diameter in each image in a sequence based on Canny edge detection.

**Figure 4:**
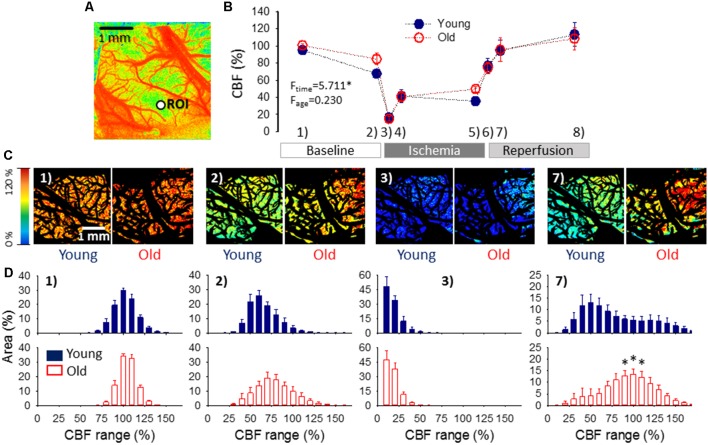
Cerebral blood flow (CBF) variation over the experimental protocol. **(A)** A pseudo-colored, representative baseline CBF map used for single-point analysis (i.e., CBF changes were extracted from a region of interest—ROI, as a function of time). **(B)** Changes of local CBF relative to baseline (100%) in the two age groups as assessed with single-point analysis. Sampling times over the experimental protocol are: (1) baseline—prior to the elicitation of the first spreading depolarization (SD); (2) baseline—between SDs; (3) ischemia—minimum CBF after ischemia onset; (4) ischemia—prior to the first ischemic SD; (5) ischemia—between SDs; (6) reperfusion—maximum CBF after reperfusion initiation; (7) reperfusion—between SDs; and (8) reperfusion—prior to the termination of the experiment. Data are given as mean ± SEM (*n* = 9/9, young/old). The statistical analysis relied on a repeated measures paradigm with age as a factor (level of significance: *p < 0.05). **(C)** Computer-generated, pseudo-colored representative CBF image pairs of a young and an old preparation used for whole field analysis. The pial vasculature is masked (black) to be excluded from the analysis targeting the cortical parenchyma. The sampling time given numerically in the upper left-hand corner corresponds to the numbering used in panel **(B)**. **(D)** Spatial flow distribution in the CBF maps, demonstrated as the relative area occupied by given CBF ranges (shown at an increment of 10%). The histograms are presented at 10% steps. Data are given as mean ± SEM (*n* = 9/9, young/old). A one-way ANOVA was used to test age-related differences (*p < 0.05).

CBF recordings obtained by LASCA (Domoki et al., 2012) were expressed relative to the baseline by using the average CBF value of the first 240 s of baseline (100%) and the recorded biological zero obtained after terminating each experiment (0%) as reference points. Local changes of CBF were first assessed by conventional single-point analysis, by positioning small ROIs (size ~19 × 19 pixels) in the CBF maps, at increasing distances from the site of SD elicitation, to extract changes in signal intensity with time (Figures 4A,B; Obrenovitch et al., 2009; (Menyhárt et al., 2017b).

Next, spatial CBF distribution over the cortical parenchyma was estimated in CBF maps, according to previously established principles (Bere et al., 2014; Clark et al., 2014). Swelling-related movement artifacts were eliminated as described above for green IOS images, to serve the spatial match of the parenchymal areas in subsequent images. The relevant parenchymal area was determined by excluding the pial vascular network and bone edges visible in the field of view (black mask in Figure 4C). The relative area occupied by pixels corresponding to given perfusion ranges (by an increment of 10% between 0% and 300%—i.e., 0–10, 11–20, 21–30%, etc…) was determined in a MatLab environment.

### Signal Processing and Data Analysis—*ex vivo* Structural Imaging

In the micro-CT images, the gross limits of the brain sample in our reconstructed volume were selected and the tissue sample was extracted. The gray values were equalized with the help of the brain sample volume’s histogram, by setting the two histogram peaks to specific grayscale values. Next, 3D Gaussian (sigma = 0.8) and median filtering [using a (3 × 3 × 3) neighborhood] was implemented. The sample was rotated to set the pial brain surface perpendicular to the z-axis of the volume by the semi-automatically calculated Euler angles. Vessels were distinguished by using a Hessian based 3D Frangi Vesselness filter implementation for MatLab with the default options. The vessel mask was produced using segmentation of the vesselness filter with the threshold of 0.00016. The volume mask was segmented from the gray value volume by Otsu-thresholding and 3D morphological closing with a sphere-shaped, 5-pixel radius structuring element. The surface mask consisted of the first pixels of the volume mask at every (x, y) location along the z-axis. Skeletonization and morphological shrink along the x- and y-axis were performed producing the vessel-skeleton mask. Vessel radius was measured as Euclidean distance between the vessel centerline and the nearest edge of the vessel mask.

The following variables were derived to characterize vascular anatomy of the cortical penetrating vasculature: (i) The volume taken up by the cerebrocortical penetrating vessels relative to the total volume of the sample was computed; (ii) The number of vascular profiles in the sample were counted in each layer of the 3D reconstruction, and averaged; and (iii) The diameter of vessels (taken as double the radius actually computed) was assessed in each layer of the sample, and averaged.

### Statistical Analysis

The software SPSS was used for statistical analysis (IBM SPSS Statistics for Windows, Version 22.0, IBM Corp.). Data are provided as mean ± standard error of the mean (SEM). When the impact of age was evaluated for a single variable (e.g., pial vascular density), an independent samples *T*-test was used. For other data sets, a one-way or two-way analysis of variance (ANOVA), or a repeated-measures model was used, dictated by the type of data set. Levels of significance were defined as *p < 0.05 and **p < 0.01. Distinct statistical methods are provided in detail in each figure legend.

## Results

### Vascular Architecture

Despite the anticipation that cerebral microvascular rarefaction should take place as rodents age (Sonntag et al., 1997; Faber et al., 2011), in this study, in both young and old rats, approximately half of the cortical surface was covered by pial vessels (Figure 1).

Micro-CT imaging of paraformaldehyde-fixed* ex vivo* tissue revealed and identified the cortical penetrating vasculature reliably (Figure 2A). On the basis of the 3D micro-CT reconstructions, the relative volume occupied by detectable microvessels was similar in the two age groups (0.27 ± 0.06 and 0.24 ± 0.02%, young and old), as well as the number of vascular profiles (per 1 mm^2^: 6 ± 1.7 and 5 ± 0.8, young and old). Likewise, the diameter of vessels appeared to be comparable in the young and old animals (15.1 ± 0.6 vs. 16.3 ± 0.7 μm, young vs. old; Figures 2B–D).

### Changes of Pial Arteriolar Diameter and CBF: Event-Free Segments of the Recording

Resting pial arteriolar diameter taken before the elicitation of the first SD obviously decreased along the branching order significantly (83 ± 6.1, 51 ± 6.9 and 31 ± 3.5 μm, for 1st, 2nd and 3rd order vessels, in the young group, *F* = 20.951, *p* < 0.0001), but was similar in the two age groups (84 ± 6.4, 54 ± 3.1 and 34 ± 2.9 μm, for 1st, 2nd and 3rd order vessels, in the old group; Figure 3B).

The induction of ischemia in the young group was followed by a prompt diameter reduction in 1st and 2nd order arterioles (to 89 ± 4.4% and 93 ± 5.4%), while the caliber of 3rd order arterioles did not change (101 ± 5.6%; Figure 3C). The diameter of 1st and 2nd order arterioles recovered in 10 min to pre-ischemic values (95 ± 2.7 and 102 ± 5.7%), and was maintained there until the initiation of reperfusion (98 ± 4.0 and 102 ± 5.1%). At the same time, 3rd order arterioles progressively dilated above their baseline tone over the ischemic period (107 ± 5.2 and 112 ± 6.9%, 10 min after ischemia onset and shortly before reperfusion). During reperfusion, pial arteriolar diameter was elevated over the baseline at all three levels of the pial arteriolar tree (111 ± 3.0, 106 ± 3.2 and 113 ± 3.1%, 1st, 2nd and 3rd order vessels; Figure 3C). A repeated-measures ANOVA test confirmed that the caliber change of various arterioles was significantly different over the experimental protocol (Figure 3C).

In the old animals, diameter changes of 1st and 2nd order arterioles were similar to that observed in the young group. However, 3rd order arterioles appeared less able to dilate under ischemia (Figure 3C). In particular, in the old rats, a marked reduction in 3rd order arteriole diameter was noted upon ischemia induction (86 ± 4.5 vs. 101 ± 5.6%, old vs. young), dilation over the first 10 min of ischemia reached a lower level (94 ± 3.7 vs. 107 ± 5.2%, old vs. young), and remained so until reperfusion onset (96 ± 5.1 vs. 112 ± 6.9%, old vs. young).

Variations of local CBF were estimated at an ROI furthest from the site of SD elicitation. As expected, the occlusion of the common carotid arteries caused a sudden drop of CBF (to 16 ± 2.6 and 15 ± 2.8% of the baseline, young and old), which settled at around 40% during the period of ischemia (41 ± 7.7 and 41 ± 6.2%, young and old). CBF recovered gradually during reperfusion from about 75% measured shortly after the release of the common carotid arteries (77 ± 8.6 and 75 ± 6.8, young and old) to approximately 110% taken prior to the termination of the experiments (114 ± 13.8 and 109 ± 12.9%, young and old). No meaningful difference was observed between young and old rats (Figure 4B).

Next, flow distribution within the field of view at selected sampling times was evaluated (Figures 4C,D). The flow ranges displayed a normal distribution at rest, and a considerable portion of the field of view fell in the perfusion ranges of 91–100% CBF (30 ± 1.8 and 34 ± 1.5% of the total area, young and old) and 101–110% CBF (24 ± 3.1 and 33 ± 3.1% of the total area, young and old). After the passage of 3 SDs, the most represented perfusion range was 51–60% CBF in the young (26 ± 3.9% of the total area), and 61–70% CBF in the old group (19 ± 4.1% of the total area), corresponding to the long-lasting oligemia that ensues SD. The flow distribution was clearly skewed to low perfusion ranges shortly after ischemia onset. In both age groups, most of the field of view was severely hypoperfused, falling in the CBF ranges of 1–10% (48 ± 9.5 and 47 ± 9.0% of the total area, young and old) and 11–20% (34 ± 4.4 and 38 ± 6.0% of the total area, young and old). Perfusion recovered to some extent between SD events, when the most represented perfusion range appeared to be 21–30% CBF (31 ± 6.6 and 25 ± 5.5% of the total area, young and old). Finally, reperfusion resulted in the widest flow distribution, and the most remarkable difference in the spatial pattern of CBF between the young and old groups. In the young animals, nearly half of the field of view was occupied by perfusion ranges between 31–70% CBF, the 41–50% CBF range being just about the most represented (13 ± 3.6% of the total area). In contrast, in the old rats, approximately half of the visible cortex was involved in the perfusion ranges of 71–110% CBF, and the 91–110% CBF range engaged the largest area (13 ± 2.3% of the total area).

### Changes of Pial Arteriolar Diameter and CBF: Response to SD

**Figure 5:**
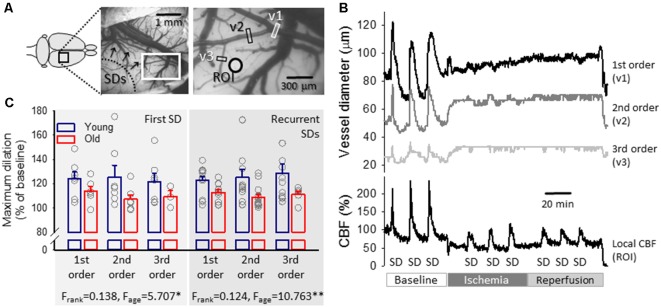
Representative traces of the variation in pial arteriolar diameter and local CBF with spreading depolarization (SD) events in a young preparation and maximum dilation of pial arterioles in response to SDs elicited during baseline. **(A)** A representative field of view over the parietal cortex of a young rat at green light illumination. Pial arteriolar segments used for the measurement of diameter changes in panel **(B)** are indicated (v1: 1st order, v2: 2nd order, v3: 3rd order arteriole). A ROI designates the origin of the local CBF trace in panel **(B)**. **(B)** The dilation of pial arterioles in response to SD events (top), with the corresponding changes in local CBF (bottom) in a representative young preparation. **(C)** Maximum dilation of 1st, 2nd and 3rd order pial arterioles in response to the first and recurrent SDs in normally perfused cortex in the two age groups. Individual values are gray symbols; bars show mean ± SEM (SD1: *n* = 6–7/3–6, young/old; recurrent SD: *n* = 11–13/5–13, young/old). A multivariate ANOVA paradigm was used for statistical analysis, considering vessel rank or age as factors (*p < 0.05 and **p < 0.01).

The representative traces in Figure 5 demonstrate that pial arterioles readily responded to SD events in the normally perfused cortex, but SD-related vascular reactivity remained obscure during ischemia. During the reperfusion phase, as a rule, vasodilation with SD was as obscure as under ischemia but was observed occasionally (in 2 young and 1 old rat). The CBF response to SDs was dominated by considerable hyperemia, which clearly stood out during the ischemia and reperfusion phases of the experiments—at the absence of reliably detectable, concomitant pial arteriolar dilation. Because diameter changes of pial arterioles were reproducible in response to baseline SDs only, the detailed analysis focused on this first phase of the experiments.

The first SD was coupled with obvious, transient vasodilation (to 124 ± 5.7, 125 ± 9.7 and 122 ± 7.0%, 1st, 2nd and 3rd order arterioles in young) and subsequent vasoconstriction (to 93 ± 2.2, 94 ± 2.1 and 95 ± 1.1%, 1st, 2nd and 3rd order arterioles in young) corresponding to considerable transient hyperemia (209 ± 7.2% CBF in young) and successive oligemia (65 ± 5.1% in young; Figure 5C). Likewise, recurrent SD events (elicited at a time when oligemia caused by the first SD was still persistent) were associated with an increase of pial arteriolar diameter (to 123 ± 3.2, 125 ± 6.5 and 129 ± 7.5%, 1st, 2nd and 3rd order arterioles in young) and marked hyperemia (198 ± 5.8% CBF in young).

Age exerted a significant impact on cerebrovascular reactivity to SD. Pial arteriolar dilation proved to be less remarkable with the first SD (1st, 2nd and 3rd order arterioles: 114 ± 4.0 vs. 124 ± 5.7, 107 ± 3.5 vs. 125 ± 9.7 and 109 ± 5.1 vs. 122 ± 7.0%, old vs. young,) and recurrent SDs (1st, 2nd and 3rd order arterioles: 113 ± 2.2 vs. 123 ± 3.2, 109 ± 2.5 vs. 125 ± 6.5 and 111 ± 2.9 vs. 129 ± 7.5%, old vs. young). Accordingly, in the old animals, the peak of hyperemia in response to SD fell behind (first SD: 174 ± 6.4 vs. 203 ± 6.8%, old vs. young; recurrent SDs: 182 ± 7.6 vs. 198 ± 6.8%, old vs. young), as reported earlier (Menyhárt et al., 2017b).

**Figure 6:**
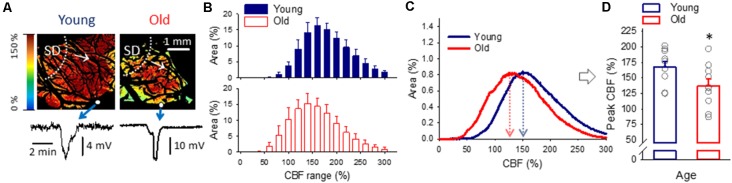
The spatial distribution of CBF at peak hyperemia in response to spreading depolarization (SD) elicited in normally perfused cortex. **(A)** Representative CBF maps of a young and an old animal demonstrate flow distribution with the passage of peak hyperemia in response to recurrent SD. The SD event was confirmed by the transient negative shift of the DC potential (bottom). Small white spheres in the images show the position of the microelectrodes implanted into the cortex. White dotted lines and arrows indicate the origin and radial direction of propagation of SD. **(B)** Relative area occupied by given CBF ranges (shown at an increment of 20%) under the passage of peak hyperemia with recurrent SDs triggered in the normally perfused cortex. Data are given as mean ± SD. A MANOVA paradigm was used to test age-related differences. **(C)** Histograms (mean for each age group) to demonstrate the relationship between CBF and surface area of the cerebral cortex with recurrent SD. **(D)** CBF at the peak of the histograms shown in panel **(C)**. Individual values are gray symbols; bars show mean ± SEM (*n* = 10/10, young/old). An independent samples *T*-test was used to statistically evaluate age-related differences (*p < 0.05).

For the characterization of spatial flow distribution in the cortex when SD-associated hyperemia ruled the field of view (Figure 6A), histograms (Figure 6C) in addition to the area representation of perfusion ranges (Figure 6B) were evaluated. While the analysis of perfusion ranges offered only trends (e.g., the most represented perfusion ranges for recurrent SDs were 141–160% in young and 121–140% in the old; Figure 6B), the histograms provided more refined information. As such, the peak of the histogram fell at a significantly lower CBF value in the old than in the young group (137 ± 11.0 vs. 167 ± 8.8%, old vs. young; Figures 6C,D), indicating a shift to the lower perfusion ranges on the CBF axis.

## Discussion

The experiments presented here have been designed to evaluate the impact of age on the hemodynamic response to SD, in an effort to understand why old age is a risk factor for accelerated lesion progression after ischemic stroke.

Even though sex differences in the epidemiology of cerebrovascular disorders are well-known (Honarpisheh and McCullough, 2019), and the susceptibility of the nervous tissue to SD may be modulated by sex (Adámek and Vyskočil, 2011), this variable has not been evaluated here.

Our previous research has shown that the magnitude of hyperemia associated with SD is decreased in the old rat brain, both in the normally perfused cortex and during ischemia/reperfusion (Menyhárt et al., 2015, 2017b), and that the amplitude of hyperemia becomes disproportionately smaller at invariable SD size in the aged brain (Menyhárt et al., 2017a, AJP Heart). Here, complementary data reveal that the dilation of pial arterioles, which is a prompt reaction to SD (Leão, 1944; Brennan et al., 2007; Ayata and Lauritzen, 2015; Menyhárt et al., 2018) is less efficacious in the aged compared to the young rat cerebral cortex, down to the smallest arterioles the resolution of our approach allowed to investigate (a diameter of ~30–35 μm at rest). The results are consistent with previous reports showing that the endothelium-dependent dilation of pial arterioles in response to the topical application of vasodilator agents (acetylcholine or bradykinin) or systemic hypercapnia is considerably smaller in aging than in young adult rodents (Mayhan et al., 1990; Balbi et al., 2015). Despite the similar age-related reduction of endothelium-dependent and SD-linked vasodilation, the cellular signaling may not necessarily be identical, since the role the endothelium may play in regulating the CBF response to SD is still uncertain (Ayata and Lauritzen, 2015). The likely cause of the age-related reduction of endothelium-linked vasodilator ability is thought to be oxidative stress (Mayhan et al., 2008). SD itself has been shown to induce oxidative stress as evidenced by the increased level of the lipid peroxidation product malondialdehyde in association with SD (Shatillo et al., 2013), but it has not been explored how oxidative stress might modulate the CBF response to SD, and how age might interfere with the signaling.

The observation concerning the pial arterioles in the present study was found relevant for the intact cortex alone because SD-related pial vasodilation remained undetectable during ischemia and reperfusion. This was not anticipated because hyperemic CBF responses were recorded repeatedly under ischemia earlier (Bere et al., 2014; Menyhárt et al., 2015, 2017b) and here with the very same SDs (Figure 5).

One might postulate that the SD-related dilation of pial vessels remained undetectable because the vessels had already reached their maximum diameter in compensation for ischemia onset or as their reaction to reperfusion. This may be an attractive explanation, but vasodilation during ischemia or reperfusion did not exceed a diameter increase of 110–115% relative to resting tone in the current experiments, while the maximum SD-coupled dilation of the same arterioles in the intact cortex (i.e., recorded over the first, baseline phase) proved to be 120–130%. These data imply that the full dilation capacity of pial arterioles was not exhausted during ischemia/reperfusion to account for the consequential lack of vasodilation with SD on ischemic background—instead, ischemia is suggested to have caused a potential impairment of arteriolar reactivity.

The dissociation of pial arteriolar diameter and local CBF variations may not be unusual. The caliber changes of pial arterioles may not strictly comply with local parenchymal CBF variations associated with SD (Ayata and Lauritzen, 2015). Indeed, the SD-coupled CBF elevation appears to correlate better with the tone adjustment of finer arteriolar branches located deeper in the cortex (Ayata and Lauritzen, 2015; Unekawa et al., 2017). The dilation of pial arterioles in response to neuronal activation has been suggested to evolve as the result of a retrograde, upstream propagation of vasodilator signals (Girouard and Iadecola, 2006). This process is thought to cause a conducted hyperpolarization of cerebrovascular smooth muscle cells or to be the result of transendothelial signaling, which originates in parenchymal microvessels (Iadecola, 2017; Longden et al., 2017). If this scheme is valid—at least in part—in the context of SD-associated vasodilation, the local hyperemia detected with LASCA in the ischemic cortex here could reflect an initial, microvascular dilation. Yet, upstream vascular conductance may have failed, and, as a result, vasodilation did not manifest at the level of the pial arterioles. Alternatively, it is also plausible that the dilation of pial arterioles with SD is caused by vasoactive substances released directly at the vessels—rather than being conducted from deeper cortical layers. Unlike parenchymal arterioles, pial arterioles are innervated by perivascular nerves originating from various ganglia (e.g., trigeminal, superior cervical), are not encapsulated by astrocytic endfeet, and are constantly bathed in cerebrospinal fluid (Ayata and Lauritzen, 2015). Due to their distinct anatomical position among cerebral vessels, pial arterioles may be selectively desensitized to vasoactive signals during ischemia, or their specific innervation may become preferentially impaired.

Because pial vasoreactivity to SD is undetectable during ischemia/reperfusion in either of the two age groups, investigating the impact of age on this variable under ischemia/reperfusion has become redundant. However, our previous data have shown that the magnitude of SD-associated hyperemia decreased with age under ischemia/reperfusion (Farkas et al., 2011; Menyhárt et al., 2017b). Here, we demonstrate that the density of the pial and penetrating vasculature were not altered in our aging rats (Figures 1, 2). These observations suggest that age-related functional impairment rather than structural maladaptation may be the underlying pathology causing the detected failure of the hyperemic response to SD.

We have made a number of additional observations with regard to aging.

First, the resting diameter of pial arterioles of the parietal cortex proved to be similar in young and aged rats (Figure 2D), indicating an intact maintenance of resting cerebral arteriolar myogenic tone. In addition, the diameter of penetrating vessels in the perfusion fixed cortical tissue was also similar across age groups. Although vessel diameter in fixed tissue may not correspond to the true resting diameter—and thus basal tone—of vessels in the living organism, it may correspond to vessel wall composition or distensibility.

Second, ischemia induction provoked compensatory vasodilation particularly in small-caliber pial arterioles in young rats, a reaction which appeared to be impaired in old animals (Figures 3C,D). The occlusion of the common carotid arteries was promptly followed by a transient drop of arteriolar diameter (probably due to the sudden fall of perfusion pressure and the transient cessation of blood flow in branches of the middle cerebral artery), before pial vasodilation occurred. The caliber regulation of the pial arterioles following ischemia onset is subject to reduced perfusion pressure, which initiates myogenic—and metabolic—responses to adjust arteriolar tone. The drop in perfusion pressure must be compensated by decreasing vascular resistance (i.e., vasodilation) in order to maintain optimal blood flow. This compensatory vasodilation (thought to be an intrinsic reaction of cerebrovascular smooth muscle cells) clearly evolved in the pial arterioles of our young rats but was less obvious in old animals (Figure 3). Production of the vasodilator nitric oxide may be an additional response to the slow re-establishment of blood flow (i.e., increasing shear stress) due to the flow re-distribution at the level of the circle of Willis after carotid occlusion. Nitric oxide production has been linked, for example, to the augmentation of collateral flow in experimental models of focal cerebral ischemia (Bonnin et al., 2019). Taken together, the results suggest that cerebral arterioles in the aging brain are less able to respond to a pressure drop or metabolic challenge optimally, in line with the insufficiency of the CBF response to SD (Menyhárt et al., 2017b) neuronal activation (Park et al., 2007; Toth et al., 2014), or hypercapnia (Balbi et al., 2015).

The endothelium is likely implicated in the arteriolar response to ischemia induction. Endothelial dysfunction in the context of cerebrovascular aging has been extensively studied (Toth et al., 2017). Oxidative stress has been recognized as a central molecular mechanism damaging endothelial function (Wang et al., 2018). Importantly, the superoxide anion is generated in the cerebral vasculature by activation of NAD(P)H oxidase, which is augmented by aging (Mayhan et al., 2008). The increased levels of superoxide anion in the aged brain quickly reacts with nitric oxide to produce peroxynitrite, and to impair endothelial nitric oxide synthase-based reactivity of cerebral arterioles. This sequence of events may have contributed to the less efficient vasoreactivity upon ischemia induction in our old rats.

Third, CBF between SD events did not differ between age groups (Figure 4B). Of note, SD is followed by a long-lasting oligemia (duration >30 min), which in our experimental paradigm did not resolve prior to the elicitation of recurrent SDs (i.e., inter-SD interval was 15 min). Post-SD oligemia is apparently mediated by 20-HETE production in vascular smooth muscle cells (Fordsmann et al., 2013) or the synthesis and release of vasoconstrictive prostanoids (Gariepy et al., 2017). On the basis of our observations, this vasoconstrictive signaling appears to be essentially preserved during the aging process.

Finally, the analysis of spatial CBF distribution revealed that a greater proportion of the aged cortex—compared to the young—was involved in higher perfusion ranges during reperfusion (Figures 4C,D). It has been proposed that CBF may exceed the resting level during reperfusion because ischemia-generated oxidative/nitrosative stress disrupts the actin cytoskeleton in cerebrovascular smooth muscle cells, which leads to diminishing myogenic tone and reduced cerebrovascular resistance (Cipolla et al., 2001; Maneen and Cipolla, 2007). The re-establishment of blood supply to the forebrain thus allows blood flow into a dilated cerebrovascular network, which causes hyperperfusion. This sequence of events suggests that, in our experiments, the ischemic-stress related dilation of the cortical microvasculature was more extensive in the old rats, possibly because of more accentuated oxidative/nitrosative stress typical of the old compared to the young nervous tissue. In turn, the hyperperfusion is thought to contribute to reperfusion injury by initiating a neuroinflammatory response and a further overproduction of reactive oxygen species (Pundik et al., 2012), thereby possibly operating a vicious cycle. Our finding raises the possibility that this pathophysiological cascade of events may become more prevalent in the aged brain.

In summary, we provide here a set of data to demonstrate that some elements of cerebrovascular structure and function are preserved while others seem to be failing with aging. As such, the density of the pial and cortical penetrating vascular network, the resting diameter of pial cortical vessels, and the degree of SD-related oligemia emerged as variables unaffected by age in our experiments, while pial arteriolar dilation in response to ischemia induction or SD were found to be reduced in the old rat brain. Also, spatial flow distribution analysis identified an age-related shift to a greater representation of higher flow ranges in the reperfused cortex.

Accumulating evidence identifies SD as a biomarker and a potent pathophysiological contributor to ischemic lesion expansion (Dreier et al., 2017; Hartings et al., 2017), possibly because of the insufficiency of the flow response or the vascular steal effect it provokes (Pinard et al., 2002; Dreier, 2011; Hoffmann and Ayata, 2013; Bere et al., 2014). Age has been identified to accelerate infarct maturation after ischemic stroke (Ay et al., 2005; Popa-Wagner et al., 2007; Faber et al., 2011). Here we provide supportive evidence, that in addition to insufficient hyperemia, the SD coupled pial vasodilation—if detectable—is compromised by old age. This impaired vasoreactivity, in turn, may contribute to a worse outcome of ischemic brain injury. Our study supports the concept that the functional impairment—rather than vascular rarefaction—may be a leading cause of the age-related deficit of the CBF response to SD, and possibly with neurovascular coupling at large. Structural abnormalities of the cerebral vasculature are also expected to exert an additional impact on flow dynamics when present. Finally, we formulate the concept that reperfusion injury may be graver in the aging compared to the young brain, because CBF during reperfusion tends to fluctuate in higher perfusion ranges, with the potential of predisposing the tissue for increased oxidative stress and inflammation.

## Data Availability Statement

The raw datasets generated and analyzed for this study can be viewed on request from the authors.

## Ethics Statement

The animal study was reviewed and approved by the National Food Chain Safety and Animal Health Directorate of Csongrád County, Hungary.

## Author Contributions

AB, ÁM, IS and EF acquired data. AB, TP, GK and EF processed and analyzed the data. TM, FB and EF designed the study. AB, ÁM, GK, IS and EF drafted the article. TM, ZK and FB revised the article critically for important intellectual content.

## Conflict of Interest

The authors declare that the research was conducted in the absence of any commercial or financial relationships that could be construed as a potential conflict of interest.
